# Green Synthesis of Silver Nanoparticle from *Anadenanthera colubrina* Extract and Its Antimicrobial Action against ESKAPEE Group Bacteria

**DOI:** 10.3390/antibiotics13080777

**Published:** 2024-08-16

**Authors:** Anastácia Nikolaos Deonas, Lucas Marcelino dos Santos Souza, Gabriel Jonathan Sousa Andrade, Jennifer Germiniani-Cardozo, Débora Dahmer, Admilton Gonçalves de Oliveira, Gerson Nakazato, José Marcelo Domingues Torezan, Renata Katsuko Takayama Kobayashi

**Affiliations:** 1 Department of Microbiology, Center for Biological Sciences, State University of Londrina, Londrina 86057-970, Brazil; anastacia.nikolaos@uel.br (A.N.D.); lucas.marcelino1@uel.br (L.M.d.S.S.); admilton@uel.br (A.G.d.O.); gnakazato@uel.br (G.N.); 2 Department of Biochemistry and Biotechnology, Center for Exact Sciences, State University of Londrina, Londrina 86057-970, Brazil; gabriel.jonathan@uel.br (G.J.S.A.); jennifer.germiniani@uel.br (J.G.-C.);; 3 Department of Animal and Plant Biology, Center for Biological Sciences, State University of Londrina, Londrina 86057-970, Brazil; torezan@uel.br

**Keywords:** biogenic Bio-AgNPs, white angico, multidrug-resistant bacteria

## Abstract

Given the urgent need for novel methods to control the spread of multidrug-resistant microorganisms, this study presents a green synthesis approach to produce silver nanoparticles (AgNPs) using the bark extract from *Anadenanthera colubrina* (Vell.) Brenan var. colubrina. The methodology included obtaining the extract and characterizing the AgNPs, which revealed antimicrobial activity against MDR bacteria. *A. colubrina* species is valued in indigenous and traditional medicine for its medicinal properties. Herein, it was employed to synthesize AgNPs with effective antibacterial activity (MIC = 19.53–78.12 μM) against clinical isolates from the ESKAPEE group, known for causing high hospitalization costs and mortality rates. Despite its complexity, AgNP synthesis is an affordable method with minimal environmental impacts and risks. Plant-synthesized AgNPs possess unique characteristics that affect their biological activity and cytotoxicity. In this work, *A. colubrina* bark extract resulted in the synthesis of nanoparticles measuring 75.62 nm in diameter, with a polydispersity index of 0.17 and an average zeta potential of −29 mV, as well as low toxicity for human erythrocytes, with a CC_50_ value in the range of 961 μM. This synthesis underscores its innovative potential owing to its low toxicity, suggesting applicability across several areas and paving the way for future research.

## 1. Introduction

Antimicrobial resistance has been the focus of much research, as major health organizations consider it one of the biggest global health issues. This concern arises from the emergence of microorganisms resistant to last-resort antibiotics, mainly caused by the inappropriate use of antibiotics in treating infections, such as the administration of sub-doses or treatment interruption. Other contributing factors include the excessive use of antimicrobials in domestic and hospital environments, the lack of public education policies regarding the correct use of antibiotics, and the overuse of antibiotics in livestock to increase animal productivity [1,2,3].

Consequently, selective pressure from the environment leads to the emergence of antimicrobial-resistant bacteria. This situation is exacerbated by the impact on various areas, including agronomy, livestock, human and animal health, and the economy, which led to the creation of the “One Health” policy. This policy aims to promote health across human, animal, and environmental contexts, as all these areas are interconnected and require joint attention [4,5].

In line with the “One Health” policy, the World Health Organization (WHO), World Organization for Animal Health (WOAH), and Food and Agriculture Organization of the United Nations (FAO) implemented, in 2015, a global program to combat antimicrobial resistance. This program, named the “Global Action Plan on Antimicrobial Resistance”, aims to achieve the following objectives: (1) promote awareness and understanding of antimicrobial resistance; (2) increase scientific knowledge on antimicrobial resistance through surveillance and research; (3) reduce the incidence of new infections through preventive measures such as basic sanitation and adequate personal hygiene; (4) improve the use of antimicrobials for people and animals; and (5) advocate for sustainable investment in new medicines, diagnostic methods, and other tools to combat antimicrobial resistance [6].

However, ongoing efforts are still insufficient, as bacterial resistance to antimicrobials was directly or indirectly related to 4.95 million deaths in 2019 [7]. Moreover, these numbers are expected to escalate. According to O’Neill [8], deaths caused by antimicrobial-resistant microorganisms are estimated to reach 10 million by 2050, surpassing the combined deaths from cancer and traffic accidents. These estimates indicate the significant impact of this problem.

Many deaths caused by antimicrobial-resistant bacteria are attributed to the group of pathogens known by the acronym ESKAPEE (originally ESKAPE, composed of *Enterococcus faecium*, *Staphylococcus aureus*, *Klebsiella pneumoniae*, *Acinetobacter baumannii*, *Pseudomonas aeruginosa,* and *Enterobacter* spp. with *Escherichia coli* included in the acronym) [9,10]. The ESKAPEE group comprises bacteria that primarily cause healthcare-associated infections (HAI) and display resistance to multiple antibiotics, demanding special attention. In 2024, the WHO published a list of priority microorganisms for research and development of new antimicrobials, encouraging investment in this area. These microorganisms were classified into critical, high, and medium-priority groups, with the ESKAPEE group included in the first two categories, critical and high priority. This reinforces the urgent need for action to combat these microorganisms [11]. Among the pathogens associated with HAIs, we can highlight, in addition to those previously mentioned, the bacterial species *Escherichia coli*. Over the last few years, some authors have reported growing concern over the increase in cases of *E. coli* pathogenic strains due to their significant role in human urinary tract infections [12,13,14]. Reports on *E. coli* pathotypes and the prevalence of resistance to antimicrobials have led to multidrug-resistant microorganisms gaining worldwide recognition as a serious health problem affecting both humans and animals, including infections caused by ESBL-producing *E. coli* [15,16,17].

Given the alarming rise of multidrug-resistant bacteria, particularly the ESKAPEE group, the search for alternatives to traditional antimicrobials is intensifying, driven by the growing concern to combat resistant microorganisms. In this context, studies on nanotechnology, bacteriophages, essential oils, plant extracts, and bacteriocins are noteworthy [18]. Within the field of nanotechnology, various studies investigate the effectiveness of nanomaterials, especially metallic nanoparticles, in combating antimicrobial-resistant microorganisms [19,20,21,22]. It is important to highlight that silver nanoparticles are the most explored and studied due to their cost-effective production and potent antimicrobial action, even against multidrug-resistant strains, including those belonging to the ESKAPEE group [21,22,23].

These metallic nanoparticles can be produced by chemical, physical, and biological methods. Naant-based synthesis (green synthesis) is gaining great interest due to several advantages over other techniques: it offers reduced production times, eliminates the need for a culture medium, is considered safe depending on the chosen plant extract, and is considered a more sustainable and environmentally friendly technology in the synthesis of nanoparticles [24,25]. This green synthesis can be carried out using microbial agents, such as fungi and bacteria, plants and their extracts, or biological agents, such as viral DNA. The use of plants, however, has attracted significant attention due to the ability of their bioactive molecules, such as amino acids, alkaloids, and phenols, to reduce metallic ions, which is vital for the biosynthetic process of nanoparticles, and the extract used in the synthesis can be obtained from different parts of the plant, such as bark, seeds, and roots, depending on availability [26]. Furthermore, some studies have demonstrated that the formation of nanoparticles with extracts can be completed in a metal salt solution in a shorter period at room temperature, depending on the plant species used, which reduces production time and costs [27]. Some examples of plants that were used in the synthesis of nanoparticles are *Malus domestica*, *Polyalthia longifolia*, *Ficus benghalensis*, and *Ananas comosus*, among others [26,27].

*Anadenanthera colubrina* (Vell.) Brenan var. colubrina plant, popularly known as “Angico”, is an endemic tree to South America, native to the Brazilian’s biomes Cerrado, Caatinga, and Atlantic Forest. It is used frequently in folk medicine to treat infections, cough, and wound healing, and in indigenous rituals [28,29,30]. Its phytocomplex, which includes a lot of secondary metabolites (approximately 56 compounds), such as phenolics (gallic acid equivalents), tannins, saponins, and flavonoids, and anadanthoflavone, which is related exclusively to this species, may be related to the pharmacological properties presented by the extract, such as antimicrobial, antioxidant, and anti-inflammatory, among others [30]. Although *A. colubrina* (Vell.) Brenan metabolic composition is quite varied, and it has a wide distribution in Brazilian territory, this plant has never been used before in the synthesis of silver nanoparticles, which corresponds to a potential to be explored. 

Considering the problem caused by antimicrobial resistance, the significant attention towards the ESKAPEE group, and the alternatives to combat these pathogens presented by nanotechnology, particularly biogenic silver nanoparticles, our study aimed at synthesizing silver nanoparticles using the bark extract from *Anadenanthera colubrina* (Vell.) Brenan var. colubrina plant and verifying the action of these nanoparticles against bacteria from the ESKAPEE group. 

## 2. Results

### 2.1. Synthesis of Biogenic Nanoparticles

**Figure 1:**
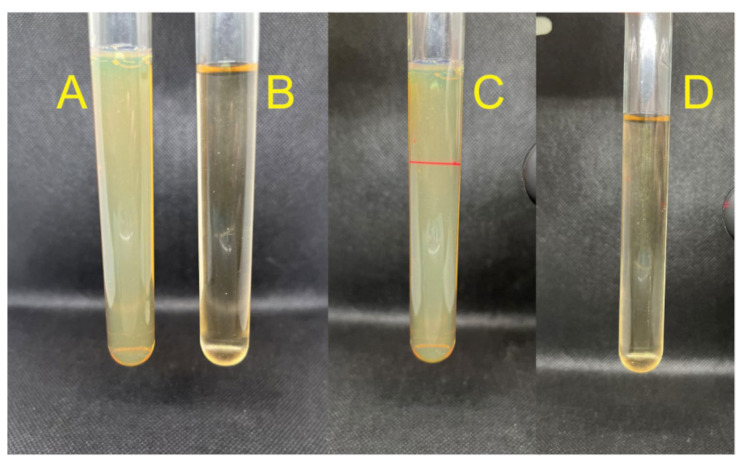
Comparison between the Bio-AgNPs biosynthesized using *A. colubrina* extract (**A**) and the extract without silver nitrate (**B**). Visualization of the Tyndall effect in the Bio-AgNPs solution (**C**), and absence of the Tyndall effect in the control extract sample (**D**).

The biosynthesis of Bio-AgNPs after 24 h resulted in a yellowish, milky appearance compared to the clear, light brown appearance of the control sample (Figure 1A,B). The colloidal nature of the sample, characterized by the presence of suspended particles, was confirmed by the Tyndall effect, in which a red laser beam was visible as it passed through the sample (Figure 1C). In contrast, when the same laser beam was applied to the control, which had the same extract concentration but without the precursor salt, the light passed through the sample without any visible alteration (Figure 1D).

### 2.2. Average Diameter Size, Zeta Potential and Polydispersity Index (PDI)

**Figure 2:**
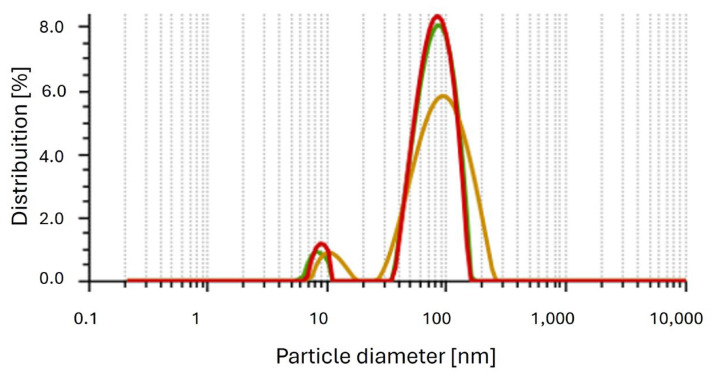
Histogram of the average size distribution of Bio-AgNPs (nm). The average diameter is 75.62 ± 0.38.

**Figure 3:**
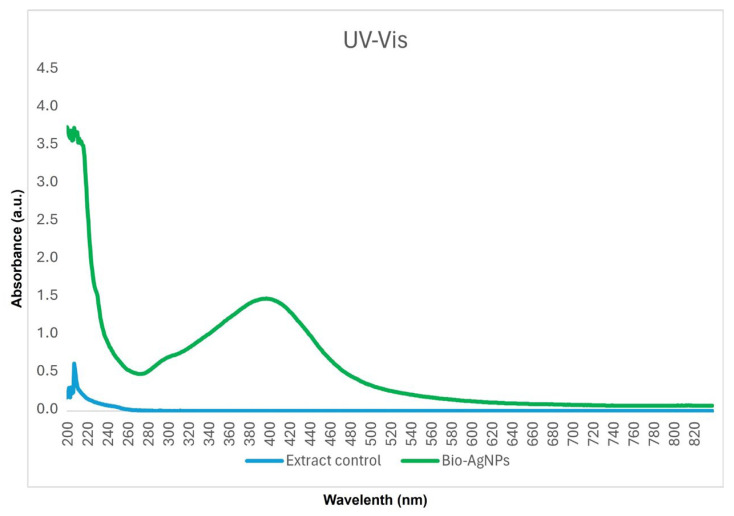
UV–Vis plasmonic spectrum of Bio-AgNPs and extract control.

The polydispersity index (PDI) reveals the homogeneity of solutions, ranging from 0 to 1. A PDI value closer to 0 indicates an ideal sample with particles of the same size, while a PDI close to 1 indicates a polydisperse sample with particles of different sizes. The nanoparticles synthesized with *A. colubrina* extract had an average diameter of 75.62 ± 0.38 nm (Figure 2), with a PDI of 0.17, indicating uniform particle size and a monodisperse sample. Additionally, the zeta potential was determined at pH 5.4 from the conversion of the electrophoretic mobility using the Smoluchowski equation with a Henry factor of 1.5. The value obtained was −29 mV. The nanoparticles synthesized with *A. colubrina* extract presented readings in absorption spectra in the range between 350 nm and 420 nm, as shown in the UV–Vis spectrum (Figure 3).

### 2.3. Fourier Transform Infrared Spectroscopy (FT-IR)

FT-IR analysis of the biosynthesized silver nanoparticles (AgNPs), compared to the control (just the bark extract), did not reveal major differences in the functional groups present in the nanoparticle coating in relation to the bark extract. However, the bands associated with these groups showed a slight shift in the spectrum, and some were overlapped by neighboring bands with an increase in intensity. In the control, the band at 3266 cm^−1^ represents hydroxyl groups common in phenolic compounds such as tannins and flavonoids, which are part of the composition of *A. colubrina* extracts [31]. The band at 1616 cm^−1^ can be associated with carbon double bonds (C=C) and the presence of carbonyl groups (C=O) conjugated to aromatic systems, typical in flavonoids [32]. The peaks at 1494 cm^−1^ are characteristic of C=C stretching in aromatic rings, indicating the presence of the mentioned phenolic compounds [33]. The bands at 1282 cm^−1^ are associated with (C-O) stretching in alcohols and ethers, characteristic of tannins and flavonoids [34], while the band at 1116 cm^−1^ is also related to C-O stretching in ethers [35]. The band at 791 cm^−1^ is coherent with out-of-plane deformations of bonds (C-H) in aromatic rings, as is the band at 616 cm^−1^, which is associated with out-of-plane deformations of C-H in benzene rings [36].

**Figure 4:**
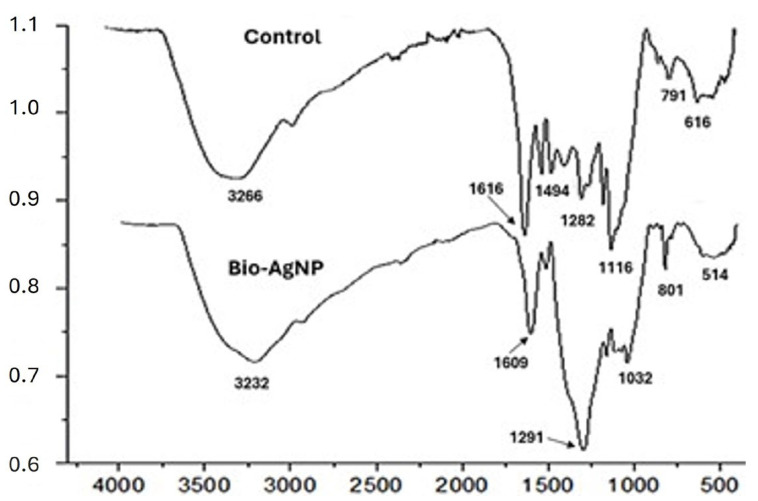
Comparison of Fourier transform infrared spectroscopy (FT-IR) spectra of the biosynthesized Bio-AgNPs and the control.

FT-IR analysis of Bio-AgNPs (Figure 4) showed the emergence of a new characteristic group (O-Ag), the maintenance of functional groups referring to (O-H), (C=C), (C-O), and the apparent absence of the band at 1494 cm^−1^. In this case, its apparent absence can be explained by the greater intensity of the band at 1291 cm^−1^ that is overlapping the neighboring bands. The bands associated with the functional groups present in both the control and the nanoparticles are slightly shifted in the spectrum, positioning themselves at 3232, 1609, and 1291 cm^−1^, respectively (Figure 4), indicating that these groups are part of the organic coating of the AgNPs. The displacement at 3232 cm^−1^ is attributed to stretching vibrations (O-H). The band at 1609 cm^−1^ corresponds to the stretching vibration (C=O) conjugated to aromatic systems typical of flavonoids, as well as the vibration (C=C) in aromatic rings. The bands at 1291 and 1032 cm^−1^ are typical of (C-O) stretching of ethers and alcohols. The band at 801 cm^−1^ is characteristic of out-of-plane vibrations of (C-H) bonds in aromatic rings. The band at 514 cm^−1^ is associated with metal-oxygen vibrations, in this case Ag-O, indicating interaction between the phenolic compounds tannins and flavonoids present in the *A. colubrina* extract and the silver nanoparticles [37].

### 2.4. X-ray Diffraction (XRD)

**Figure 5:**
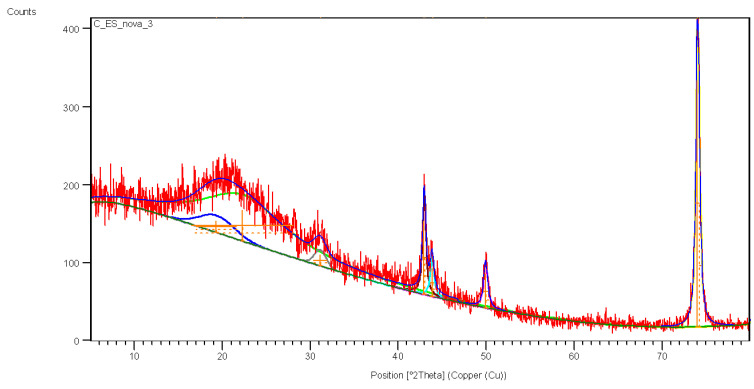
X-ray diffraction (XRD) of the control sample with *A. colubrina* bark extract.

The sample was analyzed using XRD techniques to compare the presence or absence of silver in the synthesis solution compared to the control (*A. colubrina* bark extract). The control sample did not present crystalline structures, only amorphous halos related to the phases of the plant extracts (Figure 5). The absence of organized structures in a complex sample, such as crude extracts, causes X-rays to scatter during analysis, resulting in broad peaks observed between 14° and 30° (Figure 5). 

**Figure 6:**
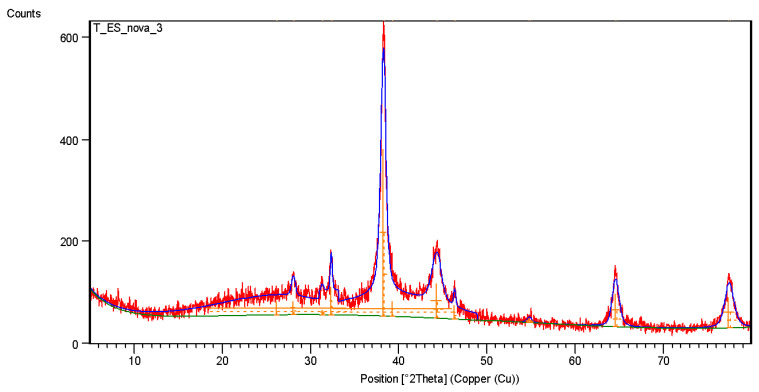
X-ray diffraction (XRD) of the Bio-AgNPs sample biosynthesized with *A. colubrina*.

In the Bio-AgNPs sample, amorphous halos were observed due to residual extract, along with peaks at 32.5° corresponding to silver and silver oxide readings, which were equivalent to 12% of the sample (Figure 6). The peaks observed in the control sample at Bragg angles of 42.9°, 43.85°, 49.9°, and 74° refer to the support where the sample was inserted to perform the analysis (Figure 5). 

Observing the diffraction pattern of the Bio-AgNPs particles, it is possible to measure a new crystallographic pattern, with peaks present at 38.01°, 44.3°, 64.4°, and 77.4° characteristic of the crystalline structure of silver nanoparticles, corresponding respectively to the (1 1 1), (2 0 0), (2 2 0), and (3 1 1) planes of the face-centered cubic arrangement [36].

### 2.5. Electron Microscopy Images

**Figure 7:**
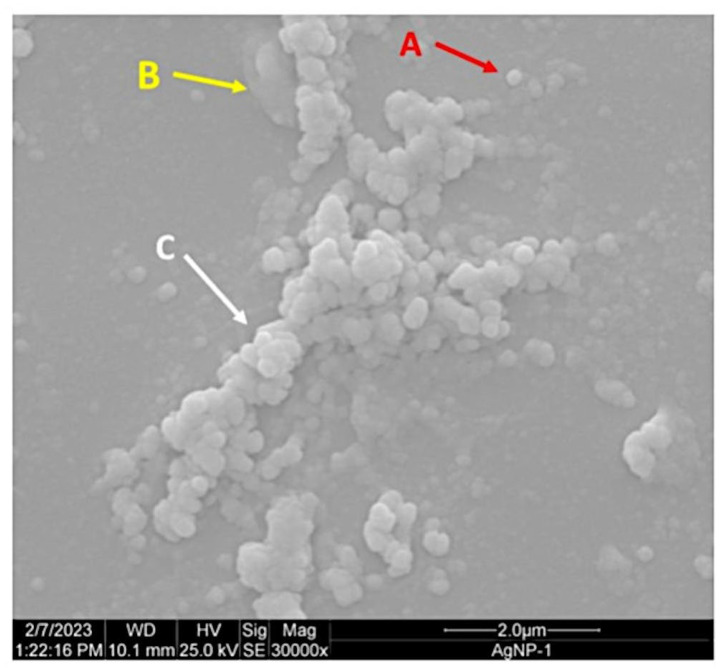
Morphology of Bio-AgNPs observed using scanning electron microscopy (SEM). Spherical morphology of isolated Bio-AgNPs (A). Organic structures from the *A. colubrina* extract (B). Aggregated arrangements of Bio-AgNPs and larger structures (C).

Scanning electron microscopy (SEM) images acquired 30 days after synthesis showed the presence of aggregated arrangements, suggesting potential stability loss due to clustering into larger structures, as well as the presence of organic structures from the *A. colubrina* extract within the sample and the spherical morphology of isolated Bio-AgNPs (Figure 7). The size of Bio-AgNPs was measured using the ImageJ software, yielding an average size of 65.78 ± 78.75 nm from SEM images. 

**Figure 8:**
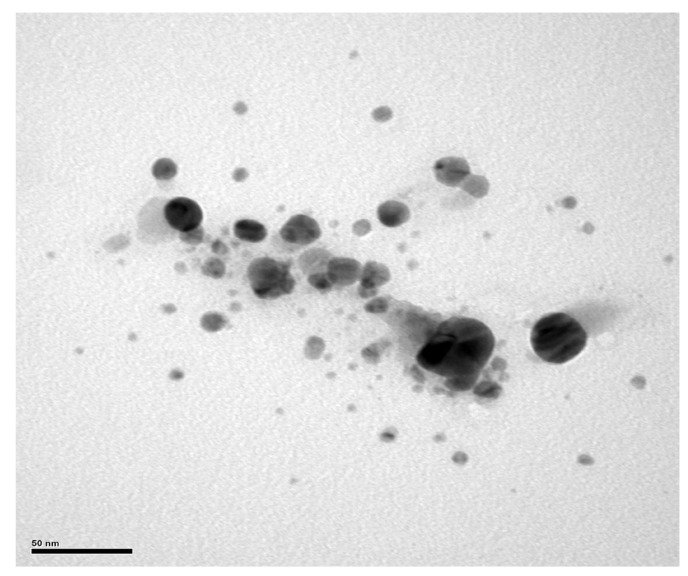
Morphological characterization of the Bio-AgNPs synthesized from *A. colubrina* with transmission electron microscopy (TEM). The micrograph shows spherical nanoparticles of Bio-AgNPs.

Transmission electron microscopy (TEM) analysis confirmed the presence of spherical Bio-AgNPs, with images showing aggregated Bio-AgNPs forming clusters with an average size of 200 nm. Moreover, TEM images captured some particles of approximately 50 nm and other smaller particles (Figure 8), although their distribution throughout the sample was limited.

### 2.6. Antibacterial Activity

The antibacterial activity of Bio-AgNPs synthesized from *A. colubrina* showed significant minimal inhibitory concentration (MIC) and minimal bactericidal concentration (MBC) values, with the lowest MIC values against Gram-negative bacteria. Negative controls using culture medium Mueller–Hinton broth (MHB) and extract at equivalent synthesis concentrations (3.1 mg/mL) and superior concentrations (1250 mg/mL) were tested showing no antibacterial activity against the tested bacteria. Additionally, the antibacterial activity of silver nitrate was evaluated for comparison with Bio-AgNPs. Sensitivity tests showed that the most susceptible strains were *A. baumannii* 141, *P. aeruginosa* 3167, and *E. cloacae* 9434, with MIC and MBC values of 19.53 μM. Both *Staphylococcus aureus* strains, ATCC 25923 and BEC 9393, showed identical MIC and MBC values of 78.12 μM and 156.25 μM, respectively (Table 1).

**Figure 9:**
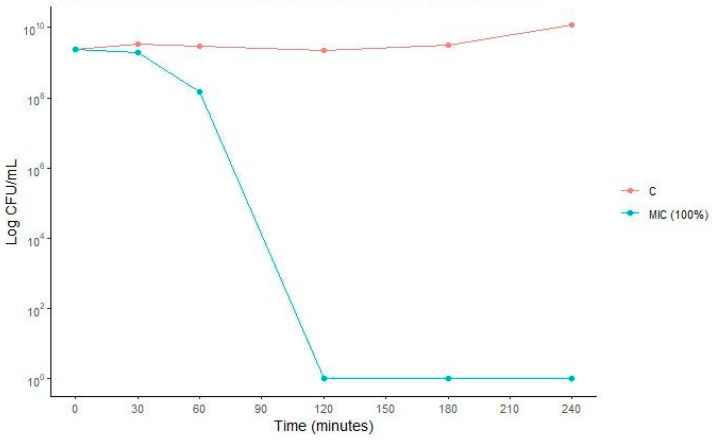
Growth kinetics and activity of Bio-AgNPs against *E. coli* ATCC 25922 at concentrations of MIC (78.12 µM). C (control) indicates the bacterial growth without antimicrobial.

*Escherichia coli* strain ATCC 25922 presented an MIC value of 78.12 μM and was subjected to a survival curve assay to elucidate the kinetics and dynamics of Bio-AgNPs action over time. This assay aimed at verifying whether Bio-AgNPs exerted a bacteriostatic or bactericidal effect. Bactericidal activity is characterized by a reduction of 3 log_10_ in bacterial concentration relative to the initial concentration of the inoculum [22]. As illustrated in Figure 9, the biosynthesized Bio-AgNPs showed bactericidal kinetics. Within just over 1 h of treatment at the MIC, bacterial concentrations dropped by 1 log_10_ compared to growth control. After 2 h of treatment, bacterial concentrations were completely reduced, indicating that the biosynthesized Bio-AgNPs exhibit bactericidal action at the MIC found in this study.

### 2.7. Hemolytic Activity

**Figure 10:**
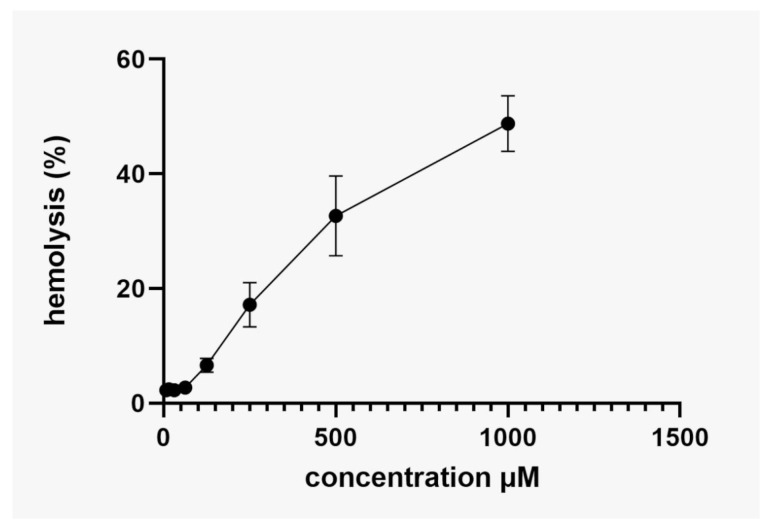
Hemolytic activity of Bio-AgNPs biosynthesized for *A. colubrina* through human erythrocytes. Values of hemolysis percentage are the mean ± standard deviation. The linear model equation used to predict the CC_50_ was y = 50.03x + 1.914 and the R-squared (R^2^).

In this study, a cytotoxicity test was carried out using human erythrocytes to verify the hemolytic capacity of Bio-AgNPs biosynthesized from *A. colubrina*. Figure 10 shows the CC_50_ of Bio-AgNPs determined by linear regression, as represented by the equation (Y = 50.03x + 1.914). The assay was performed with a maximum concentration of AgNP (1000 μM). The Bio-AgNPs biosynthesized using *A. colubrina* bark extract exhibited low hemolytic activity, with a 50% hemolysis rate at 961 μM (Figure 10). These findings suggest that the Bio-AgNPs synthesized in this work showed low toxicity for human erythrocytes once the cytotoxic concentration for these cells significantly exceeded the MIC range.

## 3. Discussion

The use of plants and plant-derived materials for treating diseases is attributed to traditional knowledge recorded over millennia by indigenous peoples and traditional communities. This practice has been encouraged and recognized by the World Health Organization since 1978 [38]. The investigation of plant-derived compounds with medicinal properties offers a basic strategy to characterize bioactive molecules and improve therapeutic approaches [39,40]. Among all the species described to date, Brazil stands out for its wide variety of plant species, distributed across six predominant biomes [41]. One of the most studied botanical families is the *Fabaceae* family, which includes *Anadenanthera colubrina*. This tree species reaches a crown height of up to 15 m, with a reddish-brown trunk and bipinnate leaves, and is found from the Caatinga to the Semideciduous Seasonal Forest [40,42,43]. The use of this plant species extends to civil construction due to the ligneous nature of its trunks [29]. *A. colubrina* is recognized for its medicinal properties, such as antimicrobial, anti-inflammatory, antioxidant, and antitumor activities [43,44]. Although this species has several therapeutic properties, some authors highlight the importance of environmental preservation owing to some beneficial properties, particularly in the seeds, in addition to warning about the necessity of replanting to minimize environmental impacts [44].

The green synthesis of Bio-AgNPs can be carried out in several ways, such as through the use of fungi, bacterial supernatant, and sand plants [45]. Studies using plants for the synthesis of Bio-AgNPs have shown nanoparticles varying from 17 nm to 50 nm, indicating that synthesis from plants can produce Bio-AgNPs of varying sizes, directly impacting their action and application [46]. Our study represents the first report of the use of *A. colubrina* bark extract to carry out Bio-AgNPs synthesis, resulting in spherical nanoparticles with an average size of 75.62 nm.

The data obtained by FT-IR analysis indicates the presence of an organic coating on the silver nanoparticles. Displacements of the bands referring to the functional groups present in the crude extract were observed, such as the bands at 3232, 1609, and 1291 cm^−1^, attributed to stretching vibrations (O-H), (C=O), and (C-O), respectively. Functional groups (C=O) conjugated in aromatic rings and (C-O) of ethers and alcohols were also detected. These characteristics are associated with phenolic compounds, including tannins and flavonoids, present in the bark extract of *A. colubrina*, as described by [31,32,33]. Therefore, this suggests that tannin and flavonoid compounds interact with nanoparticles, as their characteristic functional groups undergo a shift in the spectrum after nanoparticle synthesis. These groups probably act as stabilizing and reducing agents during biosynthesis, as evidenced by the appearance of the band at 514 cm^−1^, attributed to stretching (Ag-O). These findings are consistent with the literature, which reports the use of biocompounds for the synthesis and stabilization of silver nanoparticles [47].

Previous studies also identified the functional groups related to the *A. colubrina* bark extract as well as the interaction of biocompounds with nanoparticles, reinforcing the validity of the results presented [31,34,35,36,37]. This may have significant implications for the stability and functionality of AgNPs, potentially influencing their antibacterial and antioxidant properties, as reported in studies characterizing biosynthesized silver nanoparticles [37,48,49,50].

To determine the presence of metallic silver in the biosynthesized nanoparticles, an X-ray diffraction analysis was performed. The biological extracts exhibited no crystallinity pattern, resulting in a molecular network characterized by broad halos, which led to diffuse dispersion of the X-rays at Bragg angles [51,52]. This typical behavior was evident in the sample, which showed broad peaks between 14° and 30°. When comparing the control sample (*A. colubrina* bark extract) with silver nanoparticles (AgNP), it was possible to observe the amorphous characteristics of the crude extract and the emergence of new peaks indicative of metallic silver in the Bio-AgNPs. The presence of amorphous halos on the nanoparticles indicates their coating with biocompounds. However, the presence of crystallographic peaks at Bragg angles of 38.01°, 44.3°, 64.4°, and 77.4°, corresponding to the planes (111), (200), (220), and (311), are intrinsic characteristics of the face-centered cubic structure of silver nanoparticles. The XRD data indicate that the sample is composed predominantly of silver (88%) and a small amount of silver oxide (12%). The main peaks of the diffractogram are dominated by the silver structure, reflecting its high presence in the sample, while additional peaks correspond to the silver oxide phase. These results corroborate previous studies, confirming the presence of metallic silver in the biosynthesized nanoparticles, as well as their organic coating consisting of tannins and flavonoids from *A. colubrina* bark extract [53,54].

Despite being biogenically synthesized, the coating with plant extract around the silver nanoparticles confers unique characteristics and biological activities. According to the results obtained regarding the Tyndall effect, which indicates the scattering of laser light in the solution, and the PDI value of 0.17, both confirm the production of monodisperse Bio-AgNPs in a colloidal solution [55]. Previous research highlights that nanoparticles with a zeta potential ranging from 0 to |10| present a tendency towards instability, while those between |15| and |20| show a tendency towards stability, and values between |20| and |30| represent strong stability [56,57]. The Bio-AgNPs synthesized using *A. colubrina* extract showed an average zeta potential of −29, indicating strong stability in solution. The size, shape, composition (structure, core, and envelope), and dispersity of Bio-AgNPs reflect their physicochemical properties, in addition to influencing their bioactivity and ability to penetrate materials [58,59,60]. Studies have shown that very small particles (4 to 10 nm) are capable of permeating intact skin, potentially causing histopathological changes, while larger particles (45 nm) do not have this capacity [24,61]. 

Furthermore, the Bio-AgNPs exhibit antimicrobial action mechanisms, decreasing the properties of protection of the bacterial cell wall and affecting the cell membrane, thereby inhibiting electron transport, changing bacterial cellular function, and causing damage to genetic material [19,62,63]. Previous works that performed Bio-AgNPs synthesis from other plants have also shown excellent results against Gram-negative bacteria [19,63]. The MIC of Bio-AgNPs tested against *E. coli* strain ATCC 25922 in this and other studies using plant-synthesized Bio-AgNPs was similar, ranging around 78.12 μM. However, this work highlights low MIC values (19.53 μM) against multidrug-resistant clinical isolates such as *A. baumannii*, *P. aeruginosa,* and *E. cloacae*.

Bacteria from the ESKAPEE group are mostly resistant to conventional drugs used to control infections, underscoring their great clinical importance once these pathogens are responsible for causing numerous healthcare-associated infections [10]. According to the WHO (2024), *Escherichia coli* is among the top three pathogens on the list of critical priority due to its resistance to third-generation cephalosporins and carbapenems [11]. The pathogen *A. baumannii,* also included in this critical group, represents a serious threat to public health [11,64,65], given its challenging treatment and high risk of transmission due to its virulence mechanisms and resistance to antimicrobial drugs, leading to patient deaths and affecting the global population [66,67,68]. We can highlight that the Bio-AgNPs synthesized from *A. colubrina* were successful in controlling the growth of all microorganisms within the ESKAPEE group, precisely those listed as critical priority pathogens. Despite the absence of reports on new, effective drugs against these pathogens, continued investments in research and development for novel treatment strategies are still needed [69].

The use of Bio-AgNPs synthesized using plants represents an excellent strategy for controlling microbial growth [19,70,71]. When using plants known for their medicinal characteristics, the coat surrounding Bio-AgNPs acquires unique characteristics derived from compounds that act in the control of microorganisms and plant defense resulting from the secondary metabolism of plants [72,73,74]. *A. colubrina* bark extract has demonstrated antibacterial properties, as described in previous works [74], with MIC and MBC values ranging from 8 μg/mL to 10 μg/mL for Gram-positive and Gram-negative bacteria. This antimicrobial activity may be related to the rich phytochemical profile presented by the plant, such as the presence of phenolic compounds, which can generate metabolic instability and eliminate the enzymatic activity of proteasomes, reducing the growth rate of the microorganism and its ability to form and mature biofilms. The presence of tannins, which are part of this phenolic composition, also contributes to this activity due to the ability of these compounds to precipitate proteins, inactivating enzymes and proteins’ transportation in the cell envelope, and consequently interfering in the availability of metal ions essential to the metabolism of the microorganism [75,76]. It is important to highlight that the extraction method has a direct influence on the antibacterial compounds extracted [73,74,77]. The concentrations of the extract alone showed no activity against the tested bacteria, indicating that the antimicrobial action found in our study is exclusively attributable to the synthesized AgNPs.

Silver nitrate displayed antimicrobial action with MIC values ranging around 30 μM against *E. coli* ATCC 25922, as demonstrated in other works [78]. However, the aqueous solution of silver nitrate is reactive, presenting health risks and causing cytotoxic damage to human cells and the environment [19,22]. Optimizing AgNP synthesis using plant extracts may offer novel ways to control microorganisms, especially multidrug-resistant bacteria, directly influencing potential future applications. Thus, in this work, Bio-AgNPs were produced through green biosynthesis, which is ecologically sustainable, economically viable, and maintains exclusive characteristics justified by its synthesis. Additionally, these Bio-AgNPs possess antibacterial activity against clinically relevant Gram-positive and Gram-negative bacteria, as observed in other works [19,74,77].

The AgNP synthesis method is directly related to their cytotoxicity. Previous research using fungi for the biosynthesis of Bio-AgNPs showed lower cytotoxicity compared to other methods, such as chemical synthesis [23,25]. The CC_50_ value obtained from green synthesis using fungi was approximately 97.22 μM, indicating low toxicity [19]. Conversely, when *A. colubrina* bark extract was used for the synthesis of Bio-AgNPs, we observed even lower cytotoxicity, with a CC_50_ value of 961 μM, a concentration 12.8-fold greater than the highest MIC value (78.12 μM) obtained for the multidrug-resistant bacteria tested in this work. The highest MIC (78.12 μM) and MBC (156.25 μM) values obtained did not promote more than 5% hemolysis, which suggests that they have low toxicity and safe concentrations [79], with potential for pharmaceutical application [80]. Other studies show that the synthesis of Bio-AgNPs using plants is emerging as a promising alternative with a cost-effective approach and low impacts on human health and the environment [19,23,25,49].

## 4. Materials and Methods

### 4.1. Preparation of the Extract

The plant material used for the study was the species *Anadenanthera colubrina* (Vell.) Brenan var. colubrina. The plant sample was collected from the campus of the State University of Londrina from a single specimen located at the following geographic coordinates: 23° 19′ 26.7″ S/51° 11′ 42.0″ W, Londrina, Paraná State, Brazil. Collection was carried out using a pruning tool, and the sample was identified at the Herbarium of the State University of Londrina, under registration FUEL 56789. Moreover, registration on the National Forest Management System Platform for Genetic Heritage and Associated Traditional Knowledge (Sisgen) for recognition of genetic heritage is documented under AFCD831.

Only the outer part (the bark) was collected, avoiding pruning the specimen, ensuring minimal environmental damage or impact. The plant sample was sanitized by immersion in a solution containing distilled water and 2% sodium hypochlorite for 30 min, rinsed in running distilled water, and dried in a drying oven at 28 °C for 24 h. After drying, the sample was ground using an electric hammer mill (with Botini B55 adjustment) and stored in a glass jar with a lid, protected from light.

A decoction of the ground plant material was performed using ultrapure water at 80 °C for 1 h. The obtained extract was then sterilized by filtration using a PES syringe filter membrane (Millipore^®^, Burlington, MA, USA, 0.22 μm pore size) and stored at −4 °C for subsequent synthesis and testing.

### 4.2. Biosynthesis of Bio-AgNPs

A 100 mM stock solution of silver nitrate (AgNO_3_; molar mass 169.87 g/mol, Sigma-Aldrich^®^, Waltham, MA, USA) was prepared, filtered using a PES syringe filter membrane (Millipore^®^, 0.22 μm pore size, 33 mm diameter), and stored at −4 °C under total light protection until synthesis.

The synthesis was carried out at 37 °C under light protection for 24 h using a stock solution of plant extract (final concentration: 3.1 mg/mL) and silver nitrate (10 mM). The resulting solution had a final pH of 5.4.

### 4.3. Characterization of Bio-AgNPs

Characterizations of the obtained nanoparticles included evaluations of their morphology using scanning electron microscopy (SEM), zeta potential, polydispersity index (PDI), Fourier transform infrared spectroscopy (FT-IR), and X-ray diffraction (XRD).

The absorbance of the synthesis was evaluated by ultraviolet–visible (UV–vis) molecular absorption spectroscopy for analysis of the plasmonic band, with a reading spectrum from 250 nm to 850 nm at 5 nm intervals using a UV–vis 2600 spectrophotometer (Shimadzu, Tokyo, Japan) at the Spectroscopy Laboratory of the Multiuser Center for Research Laboratories of PROPPG/UEL.

The evaluation of the electronegativity index of the samples was determined using a zetasizer (Litesizer 500, Anton Paar, Graz, Austria), through dynamic light scattering methodology, and electrophoretic dispersion at the Multiuser Laboratory of the Federal Technological University of Paraná (UTFPR), Londrina campus.

The Bio-AgNPs were subjected to FT-IR analysis using a Michelson 30° with an automatic Peltier-controlled system for polymeric identification as well as sample freeze-dried and ground for XRD analysis composition by analyzing the molecular bonds present and functional groups present in the nanoparticle coating. A 50 mL sample of Bio-AgNPs was freeze-dried (Model L101, LIOBRAS, São Carlos, Brazil) at −56 °C and 40 mmHg. The spectrum was subjected to a resolution of 2 cm^−1^ in the range of 400–4000 cm^−1^ using a Bruker Vertex 70 spectrophotometer equipped with an ATR accessory with Ge crystal at 45° (Bruker, Ettlingen, Germany). A plant extract at the same concentration without salt was used as a control. The obtained images were processed using OriginPro 9.5 software.

The structural analyses of Bio-AgNPs were performed by XRD on a PANalytical X’Pert PRO MPD diffractometer at the X-ray Diffraction Laboratory of LARX (Multiuser Laboratory of the Pro-Rectorate for Research and Postgraduate Studies—UEL) with a voltage of 40 kV and a current of 30 mA, as well as a scanning range from 8 to 80° with an angular step of 0.04°. The samples were frozen, then freeze-dried and ground for XRD analysis.

Electron microscopy images of Bio-AgNPs were obtained using SEM (FEI Quanta 200, Quanta, Minato City, Japan) and TEM (JEM-1400, JEOL, Tokyo, Japan), following well-established methodologies and protocols for samples grown in liquid media to determine the arrangements and shapes of the synthesized Bio-AgNPs. TEM imaging was conducted at the Laboratory of Technological Innovation in the Development of Drugs and Cosmetics, COMCAP-UEM Microscopy Center, State University of Maringá, Paraná, Brazil.

### 4.4. Evaluation of the Biological Activity of Bio-AgNPs

The analyses and evaluations of the biological activity of the nanoparticles were carried out using serial microdilution methodology in broth, according to the standards described in document M07-A10 from the Clinical and Laboratory Standards Institute (CLSI) [81]. To determine the minimum bactericidal concentration, document M26-A [82] was used as a reference, with adaptations allowing the determination of the minimum inhibitory concentration (MIC) and the minimum bactericidal concentration (MBC), as well as the evaluation of the action of nanoparticles over time, assessing bacterial survival and death after treatment with inhibitory concentrations of Bio-AgNPs. Finally, the hemolytic activity of the nanoparticles was evaluated using the methodology described elsewhere [83], with some modifications, approved by the Research Ethics Committee with human beings (CAAE47661 1 15.0.0000.5231, nº. 1.268.019-UEL).

#### 4.4.1. Tested Strains

The microbiological material used in this study came from the bacterial collection of the Basic and Applied Bacteriology Laboratory, Department of Microbiology, Center for Biological Sciences, State University of Londrina, Londrina, PR. Strains stored at −20 °C in Brain-Heart Infusion (BHI) broth containing 30% glycerol were used.

Standard strains of microorganisms, including 3 standard strains from the American Type Culture Collection (ATCC): *Escherichia coli* ATCC 25922, *Escherichia coli* ATCC 8739, and *Staphylococcus aureus* ATCC 25923, were used. Multidrug-resistant clinical isolates from the University Hospital of Londrina, generously provided by Prof. Dr. Eliana Carolina Vespero and Prof. Dr. Floristher Elaine Carrara-Marroni (Clinical Microbiology Laboratory—Department of Pathology, Clinical and Toxicological Analysis of the University Hospital of Londrina), were also tested, including *E. coli* 5616, *Acinetobacter baumannii* 141, *Enterococcus faecium* 700, *Enterobacter cloacae* 9434, and *Klebsiella pneumoniae* 3978 samples. Additionally, tests were conducted with clinical isolates of *Pseudomonas aeruginosa* 3167, provided by Dr. Erika Kushikawa Saeki (Adolfo Lutz Institute—IAL of Presidente Prudente-SP) (Table 2). 

#### 4.4.2. Antibacterial Activity

To determine the minimum bactericidal concentrations, subcultures were prepared after exposure to treatments at different dilutions (ranging from 2500 μM to 19.5 μM/mL of Bio-AgNPs) in a 96-well polystyrene plate. Bacteria were exposed to treatments in each well at a final concentration of 7.5 × 10^5^ CFU/mL and incubated at 37 °C for 24 h. MIC values were determined by visual reading, checking the turbidity. MBC was assessed after an incubation period of 24 h, involving plating 10 μL from each well onto Mueller–Hinton agar (MHA), followed by another incubation for 18 to 24 h at 37 °C for CFU counting. MBC was determined as the concentration corresponding to 99.9% bacterial death compared to the bacterial growth control. All assays were performed in triplicate.

Bacterial time-kill assays were performed according to the National Committee for Clinical Laboratory Standard (NCCLS) [82], with some modifications. The *Escherichia coli* ATCC 25922 strain was cultivated in culture broth until reaching the exponential bacterial growth phase after 24 h. The bacterial suspension was adjusted to approximately 1.5 × 10^8^ CFU/mL and added to the microtubes containing Bio-AgNPs at 78.12 μM (MIC), then incubated at 37 °C. Aliquots of cultures after treatment were diluted and plated on MHA for CFU counting at different times of exposure to treatment (0.5 h, 1 h, 2 h, 3 h, 4 h, 5 h, 12 h, and 24 h). A kinetic curve of AgNP action over time was constructed as a function of incubation times.

#### 4.4.3. Hemolytic Activity Assay of Bio-AgNPs

The hemolytic activity assay of Bio-AgNPs was carried out using human erythrocytes in heparinized tubes, according to previous studies [19,83] approved by the Research Ethics Committee (CAAE 47661115.0.0000.5231, nº. 1.268.019-UEL). Erythrocytes were separated from plasma by centrifugation at 5000 rpm, 4 °C for 5 min, and subsequently diluted in phosphate-buffered saline (PBS) to a concentration of 6% (*v*/*v*). The tests were carried out in a 96-well polystyrene plate with varying concentrations of Bio-AgNPs, with the highest concentration tested being 1000 μM. PBS was used as the negative control, while 1% Triton X-100 (Sigma-Aldrich^®^) was used as the positive control, representing 100% hemolytic activity. The plate was incubated at 37 °C for 3 h, and the supernatant absorbance was read at 570 nm.

## 5. Conclusions

This work demonstrated for the first time the green synthesis of Bio-AgNPs from the bark extract of *Anadenanthera colubrina*, a tree species endemic to South America. The bark extract of *A. colubrina* resulted in the synthesis of stable spherical silver nanoparticles measuring 75.62 nm. These nanoparticles presented bactericidal action against bacterial strains and multidrug-resistant clinical isolates of the ESKAPEE group. The extract effectively coated the nanoparticles, resulting in Bio-AgNPs with unique characteristics, including excellent antibacterial activity and low toxicity to human erythrocytes (CC_50_ = 961 μM). The antibacterial action was observed against Gram-positive and Gram-negative bacteria, with the clinical isolates *Acinetobacter baumannii*, *Pseudomonas aeruginosa,* and *Enterobacter cloacae* displaying heightened susceptibility, with MIC values ranging approximately 19.53 μM, highlighting the effectiveness of plant-based synthesis for producing Bio-AgNPs. 

## 6. Patents

This work has been granted a patent by the National Institute of Industrial Property under registration number BR 10 2024 003295 0.

## Figures and Tables

**Table 1:** Antibacterial activity of Bio-AgNPs synthesized from *A. colubrina* against Gram-positive and Gram-negative bacteria, including reference and multidrug-resistant strains.

Tested Bacteria	MIC (µM)	MBC (µM)	AgNO_3_ MIC
*Enterococcus faecium* 700	39.06	156.25	31.25
*Staphylococcus aureus* ATCC 25923	78.12	156.25	62.5
*Staphylococcus aureus* BEC 9393	78.12	156.25	62.5
*Klebsiella pneumoniae* 3978	39.06	39.06	31.25
*Acinetobacter baumannii* 141	≤19.53	≤19.53	7.81
*Pseudomonas aeruginosa* 3167	≤19.53	≤19.53	7.81
*Enterobacter cloacae* 9434	≤19.53	≤19.53	31.25
*Escherichia coli* ATCC 25922	78.12	78.12	15.62
*Escherichia coli* ATCC 8739	78.12	156.25	15.62
*Escherichia coli* 5616	39.06	78.12	15.62

MIC: minimal inhibitory concentration; MBC: minimal bactericidal concentration.

**Table 2:** Antimicrobial resistance profile.

Bacterial Isolates	Resistance Profile
*Enterococcus faecium* 700	Vancomycin-resistant
*Staphylococcus aureus* BEC 9393	Methicillin-resistant
*Klebsiella pneumoniae* 3978	Carbapenem-resistant *
*Acinetobacter baumannii* 141	Carbapenem-resistant *
*Pseudomonas aeruginosa* 3167	Carbapenem-resistant *
*Enterobacter cloacae* 9434	Carbapenem-resistant *
*Escherichia coli* 5616	Carbapenem-resistant *

* Carbapenemase-producing bacteria.

## Data Availability

The original contributions presented in the study are included in the article, further inquiries can be directed to the corresponding author.
